# Testing the leadership and organizational change for implementation (LOCI) intervention in Norwegian mental health clinics: a stepped-wedge cluster randomized design study protocol

**DOI:** 10.1186/s13012-019-0873-7

**Published:** 2019-03-13

**Authors:** Karina M. Egeland, Ane-Marthe Solheim Skar, Mathilde Endsjø, Erlend Høen Laukvik, Harald Bækkelund, Aida Babaii, Lene Beate Granly, Gry Kristina Husebø, Randi Hovden Borge, Mark G. Ehrhart, Marisa Sklar, C. Hendricks Brown, Gregory A. Aarons

**Affiliations:** 1 Norwegian Centre for Violence and Traumatic Stress Studies (NKVTS), Gullhaugveien 1, 0484 Oslo, Norway; 2 Department of Psychiatry, University of California, San Diego, 9500 Gilman Drive (0812), La Jolla, San Diego, CA 92093-0812 USA; 3 Child and Adolescent Services Research Center, 3665 Kearny Villa Rd., Suite 200N, San Diego, CA 92123 USA; 4 Department of Psychology, San Diego State University, 5500 Campanile Drive, San Diego, CA 92182-4611 USA; 5 Department of Psychology, University of Central Florida, 4111 Pictor Lane, Orlando, FL 32816-1390 USA; 6 Feinberg School of Medicine, Northwestern University, 750 North Lake Shore Drive, Chicago, IL 60611 USA

**Keywords:** Implementation, Implementation leadership, Implementation climate, Leadership and organizational change for implementation (LOCI), Trauma treatment, Post-traumatic stress disorder, Outpatient clinics

## Abstract

**Background:**

Alignment across levels of leadership within an organization is needed for successful implementation of evidence-based practice. The leadership and organizational change for implementation (LOCI) intervention is a multi-faceted multilevel implementation strategy focusing on enhancing first-level general and implementation leadership while also engaging with organization upper management to develop an organizational climate for implementation. The aim of the project is to evaluate the effectiveness of LOCI in supporting the implementation of evidence-based treatment for PTSD in child- and adult-specialized mental health clinics in health trusts in Norway.

**Methods:**

The study design is a stepped-wedge cluster randomized trial with enrollment of clinics in three cohorts. Executives, clinic leaders, and therapists will be asked to complete surveys assessing leadership and implementation climate. Surveys will be completed at baseline, 4, 8, 12, 16, and 20 months. Results from surveys will be shared with executives and clinic leaders to inform the subsequent creation of tailored leadership and climate development plans for enhanced implementation. Patients will complete surveys measuring traumatic events and post-traumatic stress symptoms during the therapy process. Therapy sessions will be audio or video recorded and scored for fidelity as part of training.

**Discussion:**

This study aims to provide knowledge on how to improve leadership and organizational climate to enhance effective implementation of evidence-based treatments in mental health services.

**Trial registration:**

The study has been registrated in ClinicalTrials with ID NCT03719651.

## Introduction

Health service managers face several leadership challenges in evidence-based practice (EBP) implementation, as they need to ensure efficient use of resources and to prioritize the quality of services to be provided. Leadership has shown to be important for successful organizational change [1], achieving good climate for implementation [2], more positive attitudes toward evidence-based practices (EBPs) [3], and better patient outcomes such as patient satisfaction and quality of life [4]. However, few leadership development models or packages highlight specific strategies that organizations and leaders can use to improve the climate for implementation in their treatment teams and to improve implementation outcomes. Efforts that do not consider both contextual and individual factors likely to facilitate or hinder EBP implementation may result in poor or failed implementation, substandard service delivery, compromised patient outcomes, and decreased public health impact [5].

This project will test strategies and develop knowledge on how health care organizations and managers can lead the implementation of EBPs for post-traumatic stress disorders (PTSD) in child- and adult-specialized mental health services. Specifically, the project will test the effectiveness of the Leadership and Organizational Change for Implementation (LOCI) [5, 6] intervention in a different health care context than where it was developed. In addition, LOCI will be translated into Norwegian and delivered and facilitated by two Norwegian teams, one for youth clinics and one for adult clinics. Knowledge derived from this project has the potential to inform standards for EBP implementation so that EBPs are delivered with fidelity, thereby increasing the quality and efficiency of the health services for improved patient health outcomes.

### The leadership and organizational change for implementation (LOCI) strategy

LOCI is a 12-month empirically and theoretically based, multi-faceted, and multilevel implementation strategy that aims to improve first-level (e.g., clinic) general leadership and implementation leadership combined with the development, adaptation, and use of organizational strategies to create a positive strategic organizational climate to support EBP implementation and sustainment [6]. This study is being conducted in cooperation with the developers of LOCI through a process of adaptation for the Norwegian settings of mental health clinics with multiple health trusts. All LOCI materials were translated into Norwegian and reviewed with the US LOCI team (GAA, MGE, MS) through an iterative process of review and adaptation in regard to both materials and processes.

LOCI targets multiple levels of leadership within an organization to facilitate an aligned agenda toward EBP implementation. It is designed to strengthen leadership among first-level leaders (hereafter called LOCI leaders), who have the role of supervision of individuals providing direct services [7]. Although these leaders are in a position to facilitate EBP implementation, they may often be promoted based on clinical expertise with little support or training in effective leadership of workplace change efforts such as EBP implementation. In addition, LOCI includes leaders at higher administrative levels in order to create alignment and support for implementation across all levels of the organizational structure [8].

LOCI utilizes both the Full-Range Leadership Model (FRL) and implementation leadership as complementary leadership skills and behaviors that can be utilized to develop a positive EBP implementation climate and to effectively lead the implementation process [9–11]. The FRL is the most comprehensively researched and validated approach to leadership for individual and organizational development [12, 13]. It includes both transformational and transactional leadership. Transformational leadership is related to a leader’s ability to inspire, create a compelling vision, and motivate their employees through being a positive role model and creating a collective identity with a joint vision. Transactional leadership is related to a leader’s ability to manage and motivate their employees through appropriate rewards [14]. Appropriate use of the two dimensions is linked to effective leadership. Across several meta-analyses summarizing over a hundred studies, transformational leadership has been shown to consistently predict effectiveness outcomes across the individual and team levels of analysis [13, 15]. While the FRL addresses those leadership behaviors related to general leadership effectiveness, implementation leadership focuses specifically on how leaders can enhance implementation efforts in their organizations. The implementation leadership approach involves behaviors that fall on four dimensions: being knowledgeable about the EBP being implemented and its role in the organization, being proactive and anticipatory in problem-solving, supporting others in the implementation process, and persevering through the ups and downs of the implementation process [16].

**Fig. 1:**
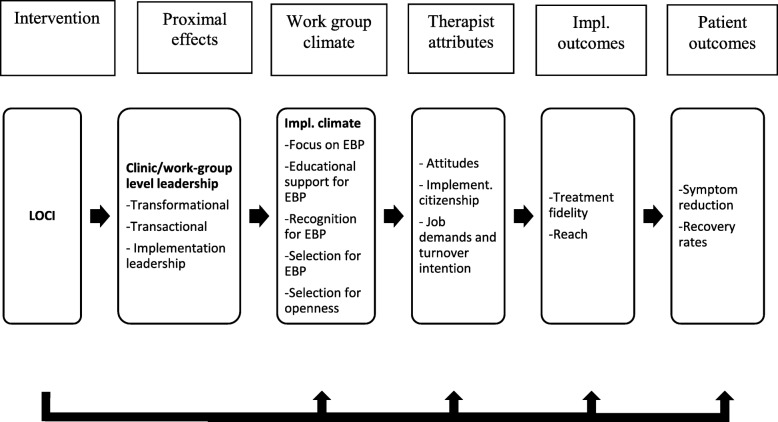
Conceptual model for the present study. Effects of LOCI on leadership, implementation climate, citizenship and job demands. Planned analyses will compare LOCI versus control on proximal and distal outcomes. Exploratory analyses will examine mediational and cross-level effects

Congruent with the emerging emphasis on mechanisms in implementation and clinical science, implementation leadership is considered to be a primary antecedent (i.e., determinant) of implementation climate (i.e., target), defined as “employees’ shared perception of the importance of innovation implementation within the organization” [11]. LOCI also builds on the work of Schein [1] utilizing climate embedding mechanisms toward enhanced implementation climate. Leaders can change and transmit the organizational culture and climate through primary embedding mechanisms, such as what the leaders and organization emphasize, value, reward, and focus on. For example, what leaders pay attention to measure and control; how they react to critical incidents; how resources are allocated for role modeling, teaching and coaching, recognition, rewards, and status; and how leaders recruit, select, and promote employees. Secondary embedding mechanisms reinforce primary mechanisms and may include organizational systems, processes, and procedures, formal statements (e.g., mission and vision) stories, and organizational rituals. LOCI also aims to improve psychological safety climate that promotes a more engaged and effective workforce [17]. It is furthermore anticipated that leadership and climate impact implementation effectiveness through employees’ actual behavior. Such behaviors include implementation citizenship behavior, defined as going above and beyond what is required in order to support the implementation of EBP [18]. The actual behavior of employees that can support the implementation of EBP can take the form of helping others, keeping informed, showing commitment to the EBP in focus, and supporting its use. Figure 1 illustrates how LOCI is hypothesized to work; the intervention will have a proximal effect on improved leadership, which will strengthen the work group climate, which will then be related to therapist conception and behavior such as clinician’s implementation citizenship behavior and sense of job demands, and ultimately to implementation outcomes, including treatment fidelity, implementation process, and patient outcomes.

Preliminary results from evaluation of LOCI have shown that the implementation strategy is feasible and acceptable; improves leader knowledge, engagement, and implementation behaviors; and has utility to improve staff-rated leadership for EBP implementation [5]. In addition, LOCI leads to improvements in staff-rated implementation climate [19]. However, similar to research on other implementation strategies, research on LOCI is still limited. It has been tested in one study [5], in addition to one ongoing randomized controlled trial [6], both in the United States. LOCI has not yet been adapted and tested in other countries. Consequently, there is a knowledge gap in the documented effect and generalizability of strategies for implementation leadership. Replication is needed, and in particular, where LOCI is conducted not by the developers, but by other researchers or service providers.

The implementation of evidence-based practices for PTSD in Norway is initiated and funded by the Norwegian Ministry of Health and Care Services and implemented by the Norwegian Center for Violence and Traumatic Stress Studies (NKVTS). The overall aims of this national initiative are that therapists’ knowledge of trauma and trauma treatment will be enhanced, patients with post-traumatic stress symptoms will receive the best evidence-supported treatments, and the knowledge and quality with which treatment methods are provided to patients will be maintained over time.

PTSD is a mental disorder that may develop after experiencing life-threatening or extreme stressful events such as accidents, assaults, or violence. The symptoms may lead to decreased functioning, social isolation, increased risk of suicide, and the development of comorbid diseases such as depression, anxiety, and substance abuse [20]. A recent study on the epidemiology of PTSD in Norway showed that the lifetime prevalence was 4.3% for women and 1.4% for men, and the mean duration of PTSD was 9 years for women and 17 years for men [21]. As a comparison, the prevalence is estimated to be higher than both lung cancer and colorectal cancer, illustrating the high prevalence of PTSD in the Norwegian population. In 2017, 6% of children in Norwegian psychiatric outpatient services received a PTSD diagnosis [22].

During the project, all therapists in the participating clinics will be trained in screening and diagnosing of PTSD. In addition, three of the most well-documented EBPs for PTSD will be implemented [23, 24]. In adult clinics, eye movement and desensitization reprocessing (EMDR) and cognitive therapy for PTSD (CT-PTSD) will be implemented. EMDR is an integrative eight-phase individual approach which is guided by the adaptive information processing model [25]. EMDR has shown to be effective for patients with PTSD in several studies [26–28]. CT-PTSD is individualized treatment using trauma memories and reactions to related triggers by imaginary and in-vivo exposure [29]. CT-PTSD has shown to be effective for patients with PTSD [30, 31]. In child and youth clinics, trauma-focused cognitive behavioral therapy (TF-CBT) [32] will be implemented. These clinics serve children aged 6–18 years and their non-offending caregiver(s) on a weekly basis over 12–15 weeks. TF-CBT is phase based, builds on cognitive behavioral principles, and incorporates principles from family therapy. Research from more than 20 randomized clinical trials (RCTs) has demonstrated that TF-CBT is effective in reducing post-traumatic stress symptoms as well as depressive symptoms (e.g., [33]).

The primary aim of the study is to investigate the effects of the LOCI implementation strategy on the implementation of EMDR, CT-PTSD, and TF-CBT in regular mental health clinical practice in Norwegian health trusts. The study’s specific aims and hypotheses are the following:

1. Test the effect of LOCI on full-range leadership and implementation leadership compared to control condition.

H1a: Implementation leadership will improve significantly more in the LOCI vs. control condition.

H1b: Full-range leadership will improve significantly more in the LOCI vs. control condition.

2. Test the effect of LOCI on clinic level implementation climate, job demands toward evidence-based practices for PTSD, and practitioner implementation citizenship behaviors compared to control condition.

H2a: Implementation climate will show significantly greater improvement in LOCI vs. control condition.

H2b: Therapists will report significantly lower job demands in the LOCI vs. control condition.

H2c: Therapists will demonstrate greater implementation citizenship in the LOCI vs. control condition.

3. Test the effects of LOCI on EBP reach.

H3: Significantly more patients will have received the EBP in the LOCI vs. control condition.

4. Test the effect of LOCI on fidelity outcomes compared to control condition.

H4a: Therapists will show greater improvements in EBP fidelity in the LOCI vs. control condition.

5. Test the effect of LOCI on patient outcomes compared to control condition.

H5a: Patients will show significantly greater improvements in symptom reduction (post-traumatic stress) and recovery rates in the LOCI vs. control condition.

6. Explore mediational and cross-level effects of leadership on climate, climate on implementation citizenship, and climate and citizenship on EBP reach (e.g., effects of clinic level climate on therapist attitudes) and test the effects of leadership strategies on implementation climate, subsequent effects on attitudes toward EBP, implementation citizenship, job demands, and turnover intention. Example hypotheses include the following:

H6a: More positive implementation climate at the clinic level will be associated with more positive therapist-level attitudes toward EBP, implementation citizenship, job demands, and turnover intention.

H6b: More positive clinic level psychological safety climate will be associated with more positive therapist-level EBP attitudes, implementation citizenship, job demands, and turnover intention.

H6c: Implementation climate will mediate the effects of leadership on attitudes toward EBP, implementation citizenship, job demands, and turnover intention.

7. Explore the need for adaptation of the LOCI intervention to the specific culture and context of Norway and each of the health trusts.

## Methods

### Design

To study the effectiveness of LOCI, a dynamic wait-list design [34], also called a stepped-wedge design [35] will be used. The stepped-wedge design is a rigorous alternative to the randomized controlled trial and parallel cluster studies. In a stepped-wedge design, all units start in their usual practice condition and clusters are randomly and sequential crossed from a control to an intervention condition. Units are typically randomized to regular time intervals (steps) wherein the units cross over to the intervention condition. As a result, at each time interval, there is a between-unit comparison of those units that are assigned to the new intervention condition and those units that remain in the usual practice condition. There is also a within-unit comparison made as the units change from the usual practice condition into the new intervention condition. In this study, units (i.e., health trusts) are randomly assigned to one of three cohorts indicating the time interval wherein that unit will participate in the LOCI intervention. This design was chosen due to the advantage of rolling out the same implementation strategy to all clinics in phases and that no clinics will be excluded from the implementation strategy.

All clinical staff in all the participating clinics will receive training in PTSD screening, and assessment and a subgroup of clinicians will receive training in PTSD treatment at the beginning of the study. The clinics will be randomized into one out of three cohorts, and the leaders will start the LOCI training at different time-periods, at 4-month intervals. Allocation will be determined by a statistician using computer-generated random number sequence. The 15 adult clinics will be randomized into three cohorts of size five based on co-located or closely working clinics (to reduce the chances of contamination), number of practitioners (< 20 or ≥ 20), and the number of practitioners per LOCI leader (< 15 or ≥ 15). For the seven health trusts encompassing the child clinics, a similar process will be used to assign them to three cohorts of size 2, 2, and 3. The variables that will be used in balancing are the total number of practices within the health trust, number of therapists trained to deliver TF-CBT, weighted centralization index, and number of municipalities served. The implementation strategy effects are determined by comparing data points in the innovation section of the wedge with those in the control section. The unit of analysis will be clinics or workgroups. Qualitative interviews with the LOCI leaders will be conducted at the end of LOCI training, in connection with the graduation, to look at the need for adaptation and documenting adaptations to LOCI.

Based on the capacity of the project group, we calculated that we could include 47 clinics. We then calculated the power based on the sample size. To calculate power, we assume the same standard deviation for differences between clinics, therapists within clinics, and within therapists. Fifteen clinics switch at time 1, 15 others at time 2, and the remaining 17 at time 3 (see Table 1). There will be no changes from time 4 to 6. The setup is longitudinal, and the same therapists are followed throughout. The answers are at the therapist level. Power calculation shows that a difference at a little below .4 standard deviations is detectable with 80% power.

**Table 1:**
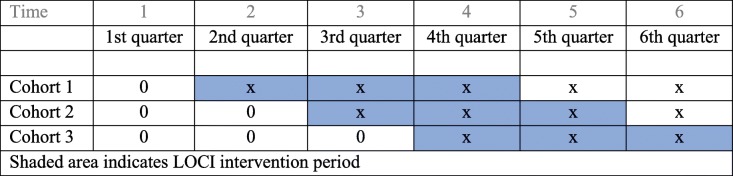
Data collection

Shaded area indicates LOCI intervention period

### Implementation framework

The project utilizes the Exploration, Preparation, Implementation, Sustainment (EPIS) conceptual framework to guide the identification of important theoretical constructs as well as their operationalization [36]. Preliminary work for this project was conducted during the exploration phase in which academic and health system partners considered both how to address PTSD, and how best to support implementation of selected EBPs. The bulk of the project will be conducted in the later three EPIS phases, and EPIS will guide tracking of progress through the phases.

### Setting

Norway consists of about 5.3 million inhabitants. Of these, 17% are immigrants or with immigrant parents, of which 7% from the European Union/European Economic Area and 4.3% are refugees. The government is responsible for the provision of treatment and the promotion of public welfare and health to everyone, on equal terms and irrespective of income. Coverage is universal and automatic for all residents, and is financed through national and municipal taxes [37]. The healthcare system is semi-decentralized and distinguishes between specialized and community care. The state is responsible for specialized care, which is administered by four Regional Health Authorities, each responsible for several health trusts that include mental health divisions consisting of both inpatient and outpatient services [38]. In 2017, 5.1% of the child population and 4.6% of the adult population received treatment in the specialized services [39].

The study will take place within Norwegian health trusts’ mental health clinics in both child- and adult-specialized services. Child and youth clinics (*Barne*- *og ungdomspsykiatrisk poliklinikk*, BUP) are outpatient clinics for children up to 18 years and their families. There are about 87 BUPs all over Norway. District psychiatric centers (DPS) include different adult outpatient and inpatient services. Within DPS, mainly general outpatient clinics will be recruited, because patients with PTSD are often referred to these clinics, including those with war and refugee experiences. There are about 75 DPSs all over Norway.

### Participants

The project will consist of four main groups of participants:

1. First-level leaders (referred to as LOCI leaders) (approximate *N* = 47) from the clinics will participate in the LOCI innovation.
2. One or more executive leaders from each health trust (approximate *N* = 23) will participate in organizational strategy meetings (described below) to support the LOCI leaders.
3. All therapists (approximate *N* = 700) in the participating clinics will be asked to participate in the training of PTSD screening. A sample of therapists (approximate *N* = 220) will be trained and supervised in PTSD treatment.
4. Patients (approximate *N* = 660) that are referred to the clinics as normal, report traumatic experiences and fulfill the criteria for PTSD will be offered to participate in the study. They will receive PTSD treatment.

### Recruitment

The project aims at recruiting 27 child and youth mental health clinics within 7 different health trusts and 20 adult mental health clinics within seven different health trusts in specialized services from all over Norway. The child and adult clinics will be recruited via the four regional health trusts, which will receive an invitation by e-mail and/or telephone. Clinics may also receive information by the project team at the NKVTS giving lectures about the project in different settings. The clinics in conjunction with the relevant health trust will decide who will participate in the LOCI training—one from each clinic or team. This will usually be the BUP leader (child) or the clinic leader (adult) but in some cases a team leader. All therapists and leaders in the participating clinics will be asked to participate in the study. They will receive advance information about the study and consent electronically prior to each survey. A sample of therapists will be trained in PTSD treatment and asked to recruit three PTSD patients each. The patients will receive advance information about the study and consent electronically (adult clinics) and in paper format (caregivers and children above 16 years old in the child clinics) prior to each survey.

Consent forms will be available in Norwegian. To accommodate other language-speaking residents, the adult patient questionnaire will be translated into English, Arabic, and Somali. The clinics will also be offered financial support for using interpreters to assist with consent forms and questionnaires.

### Inclusion and exclusion criteria

Child and adult outpatient clinics in specialized health services are included. All leaders and therapists in the clinics will be asked to participate in the surveys. Administrative employees are excluded as most of the measures will not be applicable to these staff. Staff must be employees of the participating clinics. Leaders that do not agree to participate in the leadership training (LOCI) will not be eligible to participate.

Patients (6–18 for youth clinics, 18–100 years for adult clinics) with PTSD will be asked to participate in the study by their therapist. There are no specific exclusion criteria, as the referrals should follow standard procedures within the clinics.

### LOCI intervention

The LOCI intervention consists of several components:

1. 360° assessment

A 360° assessment where the LOCI leaders, the therapists who report to them, and the executive leader are asked to complete a web-based survey focused primarily on the leadership of the LOCI leader and the implementation climate in his/her unit. Questions to therapists and the executive leaders are worded so that they are reporting on the appropriate LOCI leader. The LOCI leaders are asked questions about their own leadership, climate in their clinic, and on the implementation citizenship of their therapists.

Descriptive results from the 360° assessment are presented in an individual feedback report for each of the LOCI leaders in the LOCI condition. Each of the LOCI leaders will receive their own feedback reports during the initial and follow-up leadership trainings, and they will not be required to share it with anyone besides the research group. Feedback reports for the executives will utilize data aggregated across all clinics within the respective health trusts. Executives will be presented with their feedback reports at initial and follow-up organizational strategy meetings (see below).

#### Leadership training

**Training**

*Initial leadership training.* The LOCI intervention begins with a two-day workshop for the LOCI leaders. The workshop addresses leadership in general, with a particular focus on the full-range leadership model, transactional leadership, and implementation leadership. The LOCI leaders are challenged to share own experiences and views on leadership, and examples of leadership styles are shared and discussed. Implementation climate is also addressed, with a focus on strategies leaders can use to support implementation of EBPs. Mid-way in the workshop, at both day 1 and 2, the feedback reports from the 360° assessment will be shared individually with each of the LOCI leaders. The LOCI facilitator and the LOCI leader review the report together, identifying strengths and areas they would like to further develop. In a collaborative method, the LOCI facilitator and the LOCI leader draft that leader’s individual leadership development plan wherein goals and committed actions are detailed to facilitate enhancement in their leadership and the implementation climate of their clinic.

*Booster leadership training*. The leadership training is followed by a one-day booster session after 4 and 8 months. Before each booster session, a new 360° assessment will be completed, resulting in updated feedback reports, where the data is presented in graphs so that it is easy to see the development from the baseline assessment to the assessment at 4 and 8 months. The feedback report makes the basis for the leaders’ subsequent work with the individual leadership development plan. Organizational strategies, goals, and leadership are addressed through group discussions.

*Graduation*. At month 12, there will be a ritual to mark the completion of the program. Accomplishments are celebrated, challenges are addressed, and plans for further sustainment are shared.

**Coaching calls with first-level leaders**

The LOCI leaders will participate in weekly brief consultation calls over the phone (10–30 min) with their LOCI facilitator where the goals are to give the leaders the opportunities to strategize methods for overcoming barriers to EBP implementation, follow up on the leadership development plan, and update the leadership development plan according to the work being done and new information. Once a month, the individual calls are replaced with one-hour group consultation calls with all the LOCI leaders within the cohort.

**Organizational strategy meetings (OSMs)**

LOCI leaders and executives meet with the LOCI facilitator(s) for 2 h following the first leadership training. The first meeting is in-person. At this meeting, the LOCI leaders and executives will receive feedback from the 360° survey followed by the iterative development of an implementation climate development plan in light of the results from the survey. The subsequent meetings take place on a web conferencing platform at months 4, 8, and 12. The executive will participate in brief (15–30 min) monthly telephone coaching calls with the LOCI facilitator where the focus is to follow up on the implementation climate development plan in light of the results from the 360° survey.

### Fidelity

The LOCI developers will rate fidelity by observing the LOCI activities and documents directly or by videotaping. The Norwegian LOCI facilitators will receive regular supervision from the developers via web conferencing platform.

The therapists will audiotape (TF-CBT and CT-PTSD) or videotape (EMDR) all trauma treatment sessions from three cases through applications on iPads specifically programmed for this project. Randomly chosen therapy sessions for each therapist will be checked for fidelity through fidelity checklists. In child clinics, trained TF-CBT supervisors will do the fidelity assessments. In adult clinics, trained EMDR / CT-PTSD supervisors will assess one session on fidelity. In addition, students will assess five sessions on fidelity. The therapists will receive written fidelity feedbacks as soon as possible after the session has been videotaped or audiotaped.

#### Trauma screening and treatment training

In child clinics, the participants will receive 2 h of training in trauma and PTSS screening and assessment (CATS, see Measures). The clinics will recruit a sample of therapists (approximately 20% of the therapists) for the TF-CBT training. The therapists will receive 3 days of training and participate in 30 min weekly consultation group calls with 4–5 other TF-CBT therapists and a TF-CBT consultant (train-the-trainer certified) for 12 months. The TF-CBT consultants will conduct training and consultation. The therapists will be asked to recruit four patients each.

In adult clinics, the participants will receive half day of training in trauma and PTSD screening and assessment (TRAPS, see Measures). The clinics will be encouraged to recruit at least three therapists to both CT-PTSD and EMDR. The therapists will receive 3 days of training in one of the models. In addition, they will receive 10 h of consultation group calls divided by 2 h once a month for 5 months. Trained specialists in the interventions will conduct training and consultation. The therapists will be asked to recruit three patients each.

#### Data collection and management

Data are collected from therapists, LOCI leaders, and executives at baseline, and at 4, 8, 12, 16, and 20 months. Participants will receive invitations to complete web surveys through e-mail. Participants will not receive any compensation for their participation. The therapist and LOCI leader survey takes on average 25 min to complete, whereas the executive survey takes on average 15 min to complete. Results on leadership, implementation climate, and attitudes about EBPs will be shared with the LOCI leaders and the executive leaders as part of the LOCI feedback process.

Patients will fill out a questionnaire by iPad in connection with all therapy sessions. It takes on average 30 min to complete. Patients can receive trauma treatment even if they do not want to participate in the research. All data will be stored on the University Center for Sensitive Data and the Norwegian Centre for Research Data. Results from some of the scales will be shared with the therapist by a report function on the iPad.

Qualitative interviews will be conducted with a subsample of the LOCI leaders after the third cohort. Key questions that will be investigated include the adaption, reception, and perceived benefit of LOCI.

### Measures

#### The Implementation Leadership Scale (ILS)

ILS is a 12-item measure addressing leadership support for the usage of EBP [16]. It includes four subscales: (1) proactive leadership, (2) knowledgeable leadership, (3) supportive leadership, and (4) perseverant leadership. The scale is scored from 0 (not at all) to 4 (to a very great extent). LOCI leaders rate themselves, and their therapists and executive do the same for their LOCI leader. The scale has sound psychometric properties, with an Alpha value of .965 for the total scale [40].

#### The Multifactor Leadership Questionnaire (MLQ)

MLQ is a 36-item measure addressing transformational and transactional leadership [41]. Transformational leadership is assessed by four subscales: (1) idealized Influence (8 items), (2) inspirational motivation (4 items), (3) intellectual stimulation (4 items), and (4) individual consideration (4 items). Transactional leadership is assessed by two subscales: (1) contingent reward (4 items) and (2) active management-by-exception (4 items). The questionnaire is scored from 0 (not at all) to 4 (frequently, if not always). LOCI leaders rate themselves to which they engage in specific behaviors measured by the MLQ, and therapists do the same for their LOCI leader. The MLQ has good to excellent psychometric properties with Cronbach’s alphas ranging from .76 to .90.

#### The Implementation Climate Scale (ICS)

ICS is an 18-item measure addressing to what extent an organization supports the implementation of EBP [10]. It includes six subscales: focus on EBP, educational support for EBP, recognition for EBP, rewards for EBP, selection for EBP, and selection for openness. The scale is scored from 0 (not at all) to 4 (to a very great extent). Both the LOCI leaders, the therapists, and the executive rate the organizational climate. The ICS has excellent internal consistency and convergent and discriminant validity. The ICS has an overall Cronbach’s alpha of .91.

#### The Implementation Climate Measure (ICM)

ICM is a 6-item measure addressing the general implementation climate in the organization [42]. Consistent with the work of Klein et al. [11], it includes three subscales: what is expected, what is supported, and what is rewarded in the clinic in regard to implementation of the new innovation. The measure is scored from 0 (not at all) to 4 (often, if not always). Both the LOCI leaders, the therapists, and the executive rate the organizational climate.

#### Workload (QPS)

Thirteen items from the QPS Nordic questionnaire [43] is used to measure therapists’ coping with work (6 items) and job demands (7 items). The scale is scored from 1 (very seldom or never) to 5 (very often or always). The reliability has shown to be satisfactory and is a good instrument for assessing health-related factors at work [44].

#### The Implementation Citizenship Behavior Scale (ICBS)

ICBS is a six-item measure addressing therapist behavior that goes beyond their duty to support the implementation of EBP [18]. The scale is scored from 0 (Not at all) to 4 (Frequently, if not always). This is scored by the LOCI leader. The ICBS has demonstrated excellent internal consistency reliability (*α* = .93).

#### The evidence-based practice attitude scale (EBPAS)

EBPAS is a well-established 15-item scale to measure practitioners’ attitudes toward making use of evidence-based practices [45]. A five-point Likert scale is used to assess degree of agreement with a given statement (0 = not at all to 4 = to a very great extent). Higher mean scores indicate more favorable attitudes. The EBPAS has adequate internal consistency, and construct and content validity.

#### Turnover intentions

The staff will be asked about their intentions on staying in their job. A measure consisting of three questions will be used [46]. Responses to each item are on a seven-point scale.

#### Reach

A 15-item measure has been developed for the study, including questions regarding the therapists’ participation in training, their use of trauma assessments and PTSD treatment, and how many of their patients have received EBPs for PTSD.

#### Fidelity

*TF-CBT Brief Practice Fidelity Checklist* is an adjusted version of the TF-CBT checklist [32]. The checklist is scored as “child session,” “caregiver session,” or “child and caregiver session.” It is measured from 0 “Not Used,” 1 “Medium Extensiveness,” to 3 “High Extensiveness”. Each TF-CBT component must be addressed in the right order (if there are no therapeutic decisions on excluding a component, or to change the order of the practice), and the therapist needs to progress from one component to the next within “a reasonable time period given that the treatment should be completed within 16 sessions for usual cases and up to 20 for complex cases” [47]. Also, therapists must include parallel work with caregivers. The TF-CBT brief practice fidelity checklist will be validated during the project.

*The Treatment integrity checklist for EMDR* (used in the T-TIP study, [48]) consists of 16 items evaluating whether the therapist carries out the manual correctly (“yes” or “not relevant” = 1 point, “no” = 0 point). Maximal score is 16 points. The scale will be validated during the project.

*Cognitive Therapy for Post-Traumatic Stress Disorder*: *A Checklist of Therapist Competency* (Ehlers, not published) consists of 16 items evaluating therapists’ general and specific therapeutic skills on a range between 0 (poor) and 6 (excellent). The scale will be validated during the project.

#### Patient measures

*The Child & Adolescent Trauma Screening Questionnaire* (*CATS*), *revised version*, is used to assess trauma exposure and the frequency of all post-traumatic stress symptoms within the last 2 weeks. The first version of CATS, based on the diagnostic criteria in DSM-5, demonstrated good reliability [49]. The revised CATS is based on the diagnostic criteria in DSM-5 and ICD-11 and consists of 20 symptom items and 5 functional impairment items. A total symptom severity scale score ranging from 0 to 60 is calculated. Screening cut-off point is 15.

*The Trauma and PTSD screening* (*TRAPS*) consists of two instruments. Page one is a revised version of *Stressful Life Events Questionnaire* (*SLESQ*) [50], asking for the patients exposure of 15 different trauma events (yes or no). If the patient has experienced any of the different events, the patient will be asked to fill out page two. This consists of the *PTSD Checklist for DSM*-*5* (*PCL*-*5*) [51] and is used to assess exposure and frequency of all the post-traumatic stress symptoms within the last month (0 = not at all, 4 = a lot). Screening cut-off point is 33.

#### Data analysis

For hypotheses 1a and b; 2a, b, and c; and 4a, a mixed effects model [52] for a stepped-wedge design with multiple time points, multiple clinics (LOCI leaders), and multiple therapists per clinic leader will be estimated. All these outcomes (ILS, ICS, EBPAS, ICBS, job demands, turnover intention) are defined at the level of the leader-therapist dyad. For hypothesis 3 (reach), a similar model will be estimated, but without the therapist level since the number of patients included is not specific for individual therapists. Reach is a count variable but is planned to be estimated as continuous in this analysis. For hypothesis 5a (symptom scores), a further level is planned to include an additional level of patient within the therapist. Hypothesis 6 will be explored by causal mediation analysis [53]. Prior to substantive analyses, we will assess the data to determine if it is justified to aggregate employee responses to the first level as proposed in the nested data structure (e.g., employees nested within clinics).

## Discussion

Implementation of EBP can be challenging. In particular, the implementation of evidence-based treatment models for PTSD has been described as one of the biggest challenges in psychiatric health care [54]. This is underlined by several studies reporting that PTSD interventions are under-utilized and challenging to sustain after initial dissemination [55, 56]. Research has pointed to therapists’ concerns about using exposure-based treatment as a barrier to implementation, as they believe it can exacerbate both PTSD and comorbid symptoms [57], and that findings from clinical trials may not be representative for their patient population [58].

Leaders in the health care system are responsible for providing effective treatments in their services to a certain number of patients within a given time period. In order to increase the probability that patients will receive the most effective care for their symptoms, the leaders have to obtain effective strategies to promote the adoption of the EBP. The LOCI intervention addresses leadership in general, implementation leadership in particular, and organizational strategies in order to support the implementation of EBP and sustainment over time. Available data suggest that LOCI is effective in supporting implementation of EBP through strengthening leadership and a positive implementation climate [6]. This study will test LOCI in a Norwegian mental health setting. A stepped-wedge design will be used to investigate whether the LOCI will have an effect on leadership, the implementation climate at the clinics, and the clinic’s ability to offer trauma-focused treatments to patients with clinical levels of post-traumatic stress symptoms.

It is a strength that the design enables all clinics to receive the same implementation strategy, in addition to examining the effect of the LOCI program. However, the time gap between therapists receiving training in the PTSD treatment interventions and the leaders receiving training in the LOCI program might not be optimal for the implementation of the interventions in the last two cohorts. Moreover, even though clinics were randomized to avoid contamination between them, it might be a small chance of some contamination between clinics that are geographically close to each other.

The knowledge derived from this study can be used to support the implementation of EBPs for PTSD within mental health systems. Leaders can use strategies to develop system and organizational climates conducive to EBP implementation and sustainment. Hence, knowledge from this project can promote standards for implementation of EBPs in general through enhancing leaders’ competence in how to efficiently implement EBPs, which may increase the use of EBPs and thereby increase the quality and effectiveness of health services. The results can be transferred to health and social services in general, as the innovation can be utilized in all services that are to implement EBPs. Policy-makers and researchers are calling for more and better research in implementation science [59, 60], including the leaders’ role during the implementation process [40]. This project can hence contribute to raise an under-researched field.

## Abbreviations

CT-PTSD: Cognitive therapy for PTSD
EBP: Evidence-based practices
EMDR: Eye movement and desensitization reprocessing
EPIS: Exploration, Preparation, Implementation, Sustainment
FRL: Full-Range Leadership Model
LOCI: Leadership and organizational change for implementation
PTSD: Post-traumatic stress disorders
TF-CBT: Trauma-focused cognitive behavioral therapy
